# Treating anemia of chronic kidney disease in the primary care setting: cardiovascular outcomes and management recommendations

**DOI:** 10.1186/1750-4732-1-14

**Published:** 2007-10-02

**Authors:** Rebecca J Schmidt, Cheryl L Dalton

**Affiliations:** 1 Section of Nephrology, Department of Medicine, West Virginia University Health Sciences Center, PO Box 9165, Morgantown, WV 26506, USA

## Abstract

Anemia is an underrecognized but characteristic feature of chronic kidney disease (CKD), associated with significant cardiovascular morbidity, hospitalization, and mortality. Since their inception nearly two decades ago, erythropoiesis-stimulating agents (ESAs) have revolutionized the care of patients with renal anemia, and their use has been associated with improved quality of life and reduced hospitalizations, inpatient costs, and mortality. Hemoglobin targets ≥13 g/dL have been linked with adverse events in recent randomized trials, raising concerns over the proper hemoglobin range for ESA treatment. This review appraises observational and randomized studies of the outcomes of erythropoietic treatment and offers recommendations for managing renal anemia in the primary care setting.

## Background

**Figure 1:**
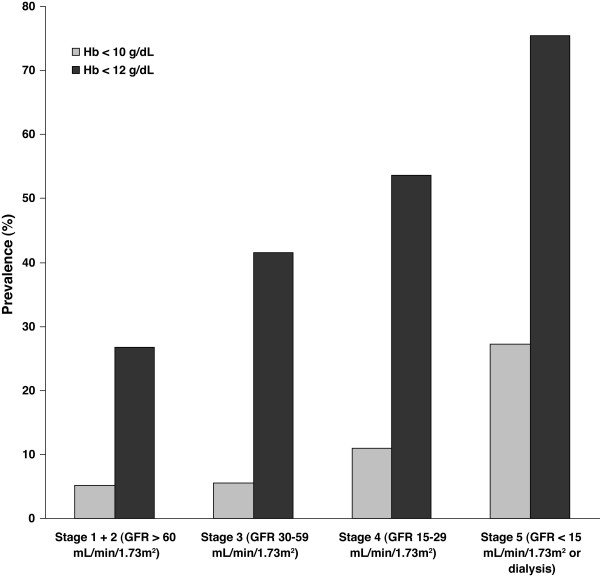
**Prevalence of anemia severity stratified by stage of chronic kidney disease**. Adapted from McClellan et al., 2004 [3].

Anemia, a common manifestation of chronic kidney disease (CKD), results primarily from inadequate renal secretion of erythropoietin [1,2]. The prevalence and severity of anemia worsen steadily as CKD advances (Figure 1) [3]. More than 30% of patients already have hemoglobin (Hb) levels <12 g/dL by Stage 3 CKD [3] when the estimated glomerular filtration rate (eGFR) falls below 59 ml/min/1.73 m^2^, and many patients develop anemia before their CKD is diagnosed [3,4]. In patients with CKD not requiring dialysis, untreated anemia increases cardiovascular risk [5-7], hospitalization [8], and all-cause mortality, [9] and diminishes health-related quality of life [10] and exercise capacity [11,12]. Heightened risk for progression of kidney failure has also been linked to untreated anemia of CKD. Thus, management of anemia throughout the CKD continuum is essential [1,2,13].

As renal disease often remains asymptomatic until eGFR falls well below 60 mL/min/1.73 m^2^, CKD, as well as its attendant anemia, remains underrecognized [14-16]. Once early CKD is diagnosed, the complexities of managing multiple comorbidities, such as uncontrolled diabetes, hypertension, hyperlipidemia, and cardiac disease, can displace clinical attention from anemia [17].

Traditionally, primary care physicians (PCPs) have been less inclined to manage anemia of CKD and have often relegated anemia treatment to a nephrologist [17-19]. However, recent claims data suggest that more than 60% of commercially insured patients with CKD-related anemia are treated exclusively by PCPs [17,19], whereas patients using Medicare [18] or Veterans' Administration services [20] are more likely to be managed by nephrologists prior to dialysis onset. Rapid increases in the end-stage kidney disease population [21] have leveled off in recent years [22]; nevertheless, the aging baby boomer generation, coupled with the epidemic of obesity and diabetes, are predicted to increase the total burden of kidney disease [23]. Nephrologists bear a formidable share of the responsibility for managing advanced CKD [24] and frequently become sole providers of primary care to dialysis patients [25]. The ratio of dialysis recipients to nephrologists is predicted to exceed 160:1 by 2010 [24]. This impending shortage highlights the need for PCP management of early CKD and its consequences.

For patients with CKD, the risk of death from cardiovascular complications exceeds the risk of progressing to renal replacement therapy [26,27]. In a managed care study [28] of 13,796 patients with eGFR values of 15–90 mL/min/1.73 m^2^, 11,278 were in Stage 3 CKD and only 777 in Stage 4, reflecting the competing risks of cardiovascular death and CKD progression. Stage 4 CKD patients of this cohort died at a higher rate every year than age- and sex-matched controls without CKD [28].

The recognition that Stage 3 CKD patients (estimated at some 6 million in the US) are more likely to die than to live long enough to reach end stage underscores the need for CKD screening as well as the importance of maintaining a high index of suspicion of occult cardiovascular disease. PCPs are thus uniquely poised to detect and treat CKD and its attendant risk factors before complications develop.

Evidence-based guidelines published by the National Kidney Foundation provide strategies for slowing the progression of kidney disease [15,16,29], yet a significant proportion of PCPs do not recognize the importance of CKD-related anemia and its treatment [17]. In a survey of 304 US physicians, only 78% of internists and only 59% of family physicians correctly identified Stage 3–4 CKD in a hypothetical case study [30]. The potential risks and benefits of treating anemia of CKD in patients not on dialysis are presented here, with particular emphasis on cardiovascular effects, the rate of CKD progression, and the implications of recent clinical trials. Fortunately, anemia is one of the most treatment-responsive complications of CKD, and its adverse physiologic sequelae can be prevented or delayed by more timely identification and management.

## Review

### Prevalence of anemia in CKD

Estimates suggest that eleven percent of adults in the US population have CKD [31] – an alarming prevalence [32,33] fueled primarily by the diabetes epidemic and an aging population that better survives longstanding heart disease [33]. US Renal Data System projections predict that nearly 500,000 people will have end-stage renal disease in 2010, compared with 431,000 in 2002 [34] and 286,000 in 1995 [35]. The cost of caring for CKD of all stages will soon exceed the cost of the Medicare renal replacement program itself [26,36]. Thus, reducing the burden of CKD and its comorbidities (including anemia) early in their course is a critical public health need.

Anemia of CKD (defined as Hb ≤12.0 g/dL) becomes increasingly prevalent as kidney function declines, ranging from approximately 27% in Stage 1 to 76% in Stage 5 (Figure 1) [3,32]. In the Prevalence of Anemia in Early Renal Insufficiency study [3], 47.7% of patients with CKD not requiring renal replacement had Hb ≤12 g/dL. Female sex (especially before menopause) [3], African-American race [3,32,37], and diabetes [3] are independent risk factors for anemia at each stage of CKD. Anemia is less common in CKD resulting from glomerulonephritis, multiple myeloma (dysproteinemia) [3], or polycystic kidney disease [38,39].

### Pathophysiology of anemia of CKD

Anemia of CKD arises primarily from a progressive failure of kidney endocrine function. Peritubular cells in the kidney cortex function as oxygen sensors controlling red cell mass. Renal tissue hypoxia triggers hypoxia-inducible factor signaling, which, in turn, up-regulates erythropoietin production [40] to stimulate division and differentiation of red cell precursors. In anemic but otherwise healthy individuals, this feedback system restores red blood cell mass and tissue oxygenation; however, in patients with CKD, one or more of these processes become impaired [16]. In early CKD, plasma erythropoietin levels may fall within the normal range, but show a blunted response to decreasing hematocrit. As CKD advances, the peritubular cells progressively diminish in number and function, producing insufficient erythropoietin to restore and maintain appropriate red cell mass [2,40].

Anemia of CKD may reflect dysregulated erythropoietin release as well as loss of peritubular cells. One hypothesis involves down-regulation of erythropoietin production in response to a decreased GFR [41]. As functioning nephron mass decreases, kidney metabolism consumes less oxygen. Because the peritubular cells are not exposed to local hypoxia, the stimulus to increase erythropoietin production is absent and anemia and peripheral hypoxia go uncorrected [41].

**Figure 2:**
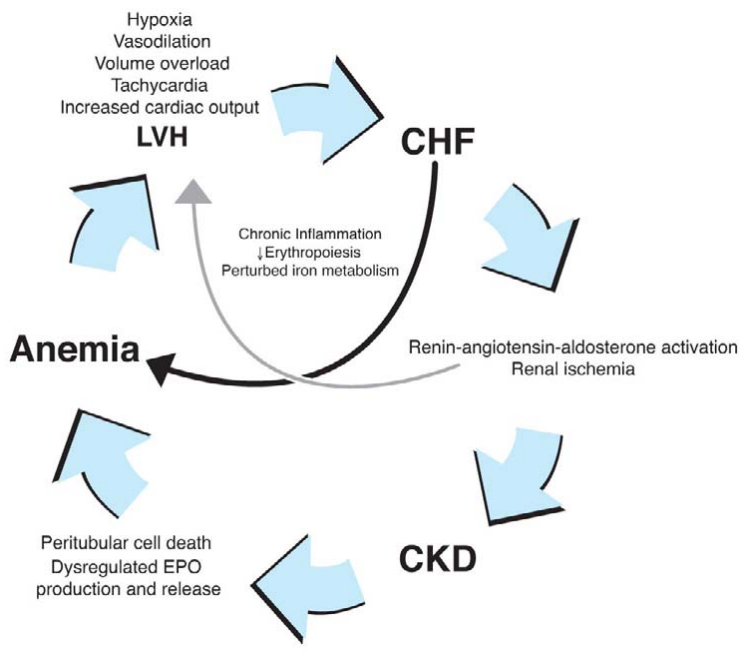
**The cardiorenal anemia syndrome**. Congestive heart failure (CHF) is a cause and consequence of CKD. First, CHF inflames the heart, liver, and vasculature, creating an influx of circulating cytokines that depress erythropoiesis and perturb iron metabolism [44]. Second, CHF directly induces kidney damage, in which GFR can deteriorate by as much as one mL/min/month [45–47]. In response to reduced cardiac output, blood pressure (and renal perfusion) is maintained by activation of the renin-angiotensin-aldosterone system. Angiotensin II-mediated renal vasoconstriction and increased metabolic demands of the kidney result in renal ischemia and ultimately tubular cell death [1]. Renal cell death in turn hastens anemia through loss of endocrine function. In addition, aldosterone-induced salt and water retention leads to an increased pre-load on the heart, which increases its rate in an attempt to increase output.

Congestive heart failure (CHF) frequently complicates CKD and adversely affects erythropoiesis. Moderate-to-severe CKD [42] has been reported in 50% of patients with CHF; conversely, approximately 40% of patients with CKD have CHF [43]. Untreated CHF may contribute to anemia of CKD both by enhancing chronic inflammation [44] and by directly inducing kidney damage [45-47] (Figure 2).

### Consequences of untreated anemia of CKD: observational studies

Among patients not requiring renal replacement, untreated anemia of CKD is strongly associated with cardiovascular [6,7,46,48-50] and renal [51,52] complications, resulting in increased hospitalizations [8,28] and mortality [51].

#### Left Ventricular Hypertrophy

The cardiovascular system compensates for low blood oxygenation by delivering a greater volume of blood to the tissues. The necessary adaptive changes – chronic vasodilation, volume and pressure overload, increased heart rate, and increased cardiac output – ultimately result in left ventricular hypertrophy (LVH) [2,7,13], whose prevalence is estimated at 39% in Stage 2 CKD, 50% in Stage 3 CKD [6,7], and 60%-74% in Stage 4 [7]. In a Canadian prospective study of patients with creatinine clearance = 25 mL/min to 75 mL/min[7], each 0.5 g/dL Hb decrease conferred a 32% increased likelihood of developing LVH [odds ratio = 1.32, 95% confidence interval (CI) 1.10 to 1.69, *P *= 0.004]. Decreasing Hb was an independent risk factor for left ventricular growth when the analysis controlled for residual kidney function [7].

#### Congestive Heart Failure

Chronic LVH and mechanical heart stress resulting from anemia contribute to development of congestive heart failure (CHF) [46]. Anemia has been described as a "mortality multiplier" in patients with comorbid CKD and CHF [53]. In the ANCHOR study [48] of 59,772 adults with CHF, 42.6% had anemia at baseline (Hb <12 g/dL for women, <13 g/dL for men). In this cohort, anemia showed a graded, independent relationship to mortality in CHF patients, the risk of death rising from 16% for 12.0–12.9 g/dL to 248% for <9.0 g/d, which compared to a reference group with Hb 13.0 g/dL to 13.9 g/dL represents adjusted hazard ratios of 1.16 and 3.48, respectively. Hb levels in relation to risk showed a J-shaped curve in this population, not all of whom had CKD; Hb levels either below 13.0 g/dL or above 17.0 g/dL were associated with increased risk of hospitalization and mortality [48].

#### Coronary Heart Disease

Anemia of CKD contributes to coronary ischemia by reducing oxygen delivery. In the Atherosclerosis Risk in Communities (ARIC) study [50], anemia independently predicted coronary heart disease in CKD patients. Participants with anemia and elevated serum creatinine (≥1.2 mg/dL in women or ≥1.5 mg/dL in men) had increased risk for coronary heart disease over 10.5 years of follow-up (relative risk = 2.74, 95% CI 1.42 to 5.28) [50]. Elevated creatinine without anemia did not significantly increase coronary risk (relative risk 1.20, 95% CI 0.86 to 1.67) [50].

#### Progression to Renal Replacement

Untreated anemia of CKD is also associated with increased risk of progression to renal replacement. In a retrospective US Veterans' Affairs cohort, each 1.0 g/dL increase in time-averaged Hb conferred a 26% reduction in risk for renal replacement (hazard ratio = 0.74, 95% CI 0.65 to 0.84) [51]. Among diabetic nephropathy patients in the Reduction of Endpoint in NIDDM with the Angiotensin II Antagonist Losartan (RENAAL) study [52], Hb <11.3 g/dL roughly doubled the risk of renal replacement onset (hazard ratio = 1.99, 95% CI 1.34 to 2.95, 3.4 years mean follow-up), and every 1 g/dL Hb decrease increased renal replacement risk by 11% [52].

### Treating anemia of CKD in patients not on dialysis: interventional studies

Several interventional studies have tested the hypothesis that treating anemia of CKD with erythropoietic agents may reduce or reverse cardiac complications and retard the rate of CKD progression (Tables 1 and 2). This hypothesis reflects not only the observational associations between untreated anemia and cardiorenal morbidity, but also the physiologic connections between anemia and cardiorenal pathology (Figure 2).

**Table 1:** Effects of subcutaneous titrated dosages of erythropoietin on cardiovascular endpoints in patients with CKD not on dialysis

**Reference (study design)**	**Level of renal function; study duration**	**No. of pts**	**Treatment regimen**	**Endpoint**	**Outcome**
**Left ventricular hypertrophy**					

Ayus et al. [54] (uncontrolled)	CrCl 10–30 mL/min (diabetic) or 20–40 mL/min (nondiabetic); 6 mo	40 (Hb <10 g/dL)	EPO to 12 g/dL	Change-from-baseline LVMI	In anemic pts, LVMI decreased vs baseline (142 vs 157 g/m^2^;*P *= 0.007) as Hb increased from 9.1 to 11.3 g/dL (*P *= 0.001).
		61 (Hb>10 g/dL)	Standard care		
CREATE study [61] (r)	GFR 15–35 mL/min/1.73 m^2^, Hb 11.0–12.5 g/dL;3 yrs	300	EPO to 13–15 g/dL	Composite of 8 cardiovascular events (primary), LVMI (secondary)	Baseline LVMI: high-Hb group, 120.3 ± 35.0 g/m^2^ low-Hb group, 118.0 ± 34.3 g/m^2^ Change at year 1: High-Hb group, -4.6 g/m^2^ Low-Hb group, -3.3 g/m^2^; *P *= 0.59 Change at year 2 High-Hb group, -6.4 g/m^2^ Low-Hb group, -7.8 g/m^2^
		300	EPO to 10.5–11.5 g/dL		
Levin et al. [56] (r)	24 mo	78	Early EPO to Hb 12–14 g/dL	Mean change-from-baseline LVMI	Mean LVMI change from baseline: early EPO, +0.37 g/m^2^ deferred EPO, +5.21 g/m^2^
		58	Deferred EPO to 9.0–10.5 g/dL		
Roger et al. [57] (r, mc, uncontrolled)	CrCl 15–50 mL/min, Hb 11.0–12.0 g/dL (in women) and 11–13 g/dL (in men);2 yr or until dialysis	75	EPO to Hb 12–13 g/dL	Mean change-from baseline LVMI	No statistically significant between group changes in LVMI over 2 years. Based on per-protocol Hb levels: Change from baseline LVMI: Low Hb group +14 g/m^2^ High Hb group -1 g/m^2^.
		80	EPO to Hb 9–10 g/dL		

**Congestive heart failure**					

Mancini et al. [77] (r)	SrCr<2.5 mg/dL, NYHA functional class III-IV, Hct<35%;3 mo	15	EPO 15 000–30 000/wk	Blood and exercise parameters	Changes from baseline: EPO group: Peak VO_2 _11.0 to 12.7 mL·min^-1^·kg^-1^, *P *< .05 Exercise duration 590 to 657s, *P *< 0.004 Placebo group: no significant changes
		8	Placebo		
Silverberg et al. [45] (retrospective)	Mean NYHA 3.66, SrCr 2.6 mg/dL, Hct 30%, Hb 10 g/dL;>6 mo	26	EPO + IV iron to Hb 12 g/dL	NYHA functional status, LVEF, healthcare utilization	Changes from baseline: Functional status 3.7 to 2.7, *P *< 0.05 LVEF 28% to 35%, *P *< 0.001 No. of hospitalizations/pt 2.7 to 0.2, *P *< 0.05).
Silverberg et al. [47] (r)	NYHA class III-IV, LVEF ≤40%, Hb 10–11.5 g/dL, 50% with CKD; 8.2 mo	16	EPO + IV iron to Hb ≥12.5 g/dL	NYHA functional status, LVEF, furosemide requirements, healthcare utilization	Changes from baseline: EPO + iron group: NYHA class, 42.1% improvement LVEF: +5.5% Length of hospitalization: -79% Standard care group: NYHA class: 11% worsening LVEF: -5% Length of hospitalization: +58%
		16	Standard care		
Silverberg et al. [46] (nr)	NIDDM or no NIDDM plus severe CHF, GFR decline >1 mL/min/mo; 11.8 mo	84 (NIDDM)	EPO to Hb 12.5 g/dL + IV iron PRN	NYHA functional class; VAS for fatigue and breathlessness; LVEF	Changes from baseline: NIDDM pts: NYHA functional class 35% improvement LVEF +7% non-NIDDM pts: NYHA functional class: 32% improvement LVEF +12% No statistically significant differences between NIDDM and non-NIDDM.
		95 (no NIDDM)			
Silverberg et al. [78] (nc)	Symptomatic CHF despite optimal therapies, Hb<12 g/dL,91% had CKD (CrCl <60 mL/Min);20.7 mo	78	EPO + PRN IV iron to Hb 13 g/dL	NYHA functional class, LVEF, healthcare utilization	Changes from baseline: (all *P *< 0.01). NYHA class 2.5 (vs 3.7), LVEF 37% (vs 33%); No. of hospitalizations 0.7/year (vs 2.7/year)

**CrCl **= creatinine clearance; **db **= double-blind; **EPO **= epoetin; **GFR **= glomerular filtration rate; **Hct **= hematocrit; **IV **= intravenous; **LOHS **= length of hospital stay; **LVEF **= left ventricular ejection fraction; **LVMI **= left ventricular mass index; **mc **= multicenter; **mo **= months; **nc **= noncomparative; **NYHA **= New York Heart Association; **PLA **= placebo; **PRN **= as required; **pts **= patients; **r **= randomized; **SrCr **= serum creatinine; **VAS **= visual-analog scale; **wk **= week.

**Table 2:** Effects of subcutaneous titrated dosages of erythropoietin in patients not on dialysis on progression to renal replacement therapy

**Reference (study design)**	**Level of renal function; trial duration**	**No. of pts**	**Treatment regimen**	**Endpoint**	**Outcome**
Dean et al. [55] (retrospective)	eGFR 30–59 mL/min/1.73 m^2 ^(n = 71) eGFR <29 mL/min/1.73 m^2 ^(n = 51) Hb = 10–11.9 g/dL or 8.0–9.9 g/dL	122 Only pts with ≥8 EPO doses included	EPO (Hb targets, doses not specified	Change in least-squares slope of inverse serum creatinine clearance vs time before and after EPO	Baseline eGFR 30–59 ml/min/1.73 m^2^: Pre-EPO rate, dL/mg/yr: -0.0981 (95% CI, -0.12, -0.07) Post-EPO rate, dL/mg/yr: -0.0692 (95% CI, -0.09, -0.04) Weighted mean difference, dL/mg/yr: 0.0454 (95% CI, 0.0150, 0.0757) Baseline eGFR <29 ml/min/1.73 m^2^: Pre-EPO rate, dL/mg/yr: -0.0899 (95% CI, -0.12, -0.06) Post-EPO rate, dL/mg/yr: -0.0416 (95% CI, -0.06, -0.02) Weighted mean difference, dL/mg/yr: 0.0493 (95% CI, 0.0272, 0.0679) Overall: Pre-EPO rate, dL/mg/yr: -0.0937 (95% CI, -0.11, -0.08) Post-EPO rate, dL/mg/yr: -0.0569 (95% CI, -0.07, -0.04) Weighted mean difference, dL/mg/yr: 0.0475 (95% CI, 0.0272, 0.0679) *P *< 0.05
CREATE study [61] (r)	eGFR 15–35 mL/min/1.73 m^2^, Hb 11.0–12.5 g/dL;3 yrs	300	EPO to 13–15 g/dL	Time to dialysis (secondary) eGFR also assessed	Change of mean eGFR from baseline: Year 1: High-Hb group -3.6 ± 6.7 mL/min Low-Hb group -3.1 ± 5.3 mL/min Time to dialysis was significantly shorter in high-Hb group (*P *= 0.3)
		300	EPO to 10.5–11.5 g/dL		
Gouva et al. [58] (r, mc)	SrCr 2–6 mg/dL (eGFR not given), Hb 9.0–11.6 g/dL;22.5 mo	45	Early EPO to Hb≥3 g/dL	Composite of doubling of baseline SrCr, renal replacement or death	Composite endpoint: Early EPO pts, 29% Deferred EPO pts, 53%; *P *= 0.0078 Renal replacement: Early EPO pts, 22% Deferred EPO pts, 42%; *P *= 0.011
		43	Deferred EPO when Hb<9.0 g/dL		
Jungers et al. [59] (c-cs)	Predialysis (CrCl ≤15 mL/min) pts (eGFR not given); 24 mo	20 (Hb<10 g/dL)	EPO to 11.5 g/dL	Change-from-baseline rate of decline in creatinine clearance, time to dialysis	Rate of change in creatinine clearance, mL/min/1.73 m^2^/month: EPO group: Baseline -0.36 ± 0.16 End of study -0.26 ± 0.15 (*P *< .05) Standard care group: Baseline -0.55 ± 0.48 End of study -0.57 ± 0.44 Time to dialysis: EPO group 16.2 ± 11.9 mo Standard care group 10.6 ± 6.1 mo (*P *< 0.01).
		43 (Hb>10 g/dL)	Standard care		
Kuriyama et al. [79] (r)	SrCr 2–4 mg/dL (eGFR not given), Hematocrit<30%;28 mo median follow-up	31	Standard care	Doubling of baseline SrCr	Doubling of baseline SrCr EPO, 52% of pts Standard care, 84% of pts Nonanemic control, 60% of pts Progression to dialysis: EPO, 33% of pts Standard care, 65% of pts (*P *= 0.008) Nonanemic control, 37% of pts
		42	EPO to Hct 33–35%		
	SrCr 2–4 mg/dL, Hematocrit>30%;28 mo median follow-up	35	Nonanemic control		
Rossert et al. [66] (r, mc, uncontrolled)^a^	Iothalamate GFR 25–60 mL/min; 40 mo	108	EPO for Hb 14–15 g/dL (men) and 13–14 g/dL (women)	Change-from-baseline GFR as estimated by iohexol clearance	GFR change: High Hb group -0.058 mL/min/1.73 m^2^ Low Hb group -0.081 mL/min/1.73 m^2^. No significant difference between groups
		133	PRN EPO for Hb 11–12 g/dL		
Silverberg et al. [46] (nr)	Cockcroft-Gault eGFR decline >1 mL/min per mo; 11.8 mo	84 (NIDDM)	EPO to Hb 12.5 g/dL + IV Iron PRN	SrCr and CrCl (secondary endpoints)	GFR decline halted in both groups
		95 (no NIDDM)			

**a **Because of labeling changes for EPO, this study terminated after 7.4 and 8.3 months of maintenance in the high and low Hb group, respectively.

**c-cs **= case-control study; **CHF **= congestive heart failure;**CrCl **= creatinine clearance; **db **= double-blind; **EPO **= epoetin; **(e)GFR **= (estimated) glomerular filtration rate; **Hct **= hematocrit; **mc **= multicenter; **IV **= intravenous; **mo **= months; **NIDDM **= noninsulin-dependent diabetes mellitus; **nr **= nonrandomized; **PLA **= placebo; **PRN **= as required; **pts **= patients; **r **= randomized; **SrCr **= serum creatinine; **wks **= weeks.

Early interventional studies [47,54-59] supported the notion that treating anemia with erythropoietic agents improves cardiac and renal prognosis. Unexpectedly, the recent randomized controlled trials Correction of Hemoglobin and Outcomes in Renal Insufficiency (CHOIR) [60] and Cardiovascular Risk Reduction by Early Anemia Treatment with Epoetin (CREATE) [61] showed unforeseen increases in cardiovascular events [60] and dialysis initiation [61] among patients assigned to the highest Hb targets, prompting reexamination of the optimal targets and appropriate recipients of erythropoietic therapies.

The primary outcome of the US-based CHOIR study (N = 1432), which enrolled CKD patients with eGFR values of 15 to 50 mL/min/1.73 m^2 ^and Hb levels <11 g/dL, was a composite of death, myocardial infarction, hospitalization for heart failure, or stroke [60]. Patients were allocated to a high-Hb group (target, 13.5 g/dL) or a low-Hb group (target, 11.3 g/dL); Hb goals were achieved and maintained by titrated dosages of epoetin alfa. The trial was stopped after a mean follow-up of 16 months, when the primary outcome was reached by more patients in the high-Hb than low-Hb group (17.5% vs. 13.5%, *P *= 0.03). Significantly more patients in the high-Hb group reported histories of hypertension (95.8% vs 93.2%; *P *= 0.03) or coronary artery bypass grafts (17.4% vs 13.5%; *P *= 0.05) at baseline, suggesting an uneven baseline cardiovascular risk burden between the groups.

The design of the international CREATE study (N = 603) [61] was similar to CHOIR. Patients with eGFR values of 15.0 mL/min/1.73 m^2 ^to 35.0 mL/min/1.73 m^2 ^and Hb levels of 11 g/dL to 12.5 g/dL were randomized to receive early epoetin beta therapy to an Hb target of 13 g/dL to 15 g/dL or deferred epoetin beta therapy initiated when Hb levels fell below 10.5 g/dL. The primary endpoint was a composite of eight cardiovascular events. During an average follow-up of three years, the likelihood of a first cardiovascular event was not statistically different in the high-Hb group than in the low-Hb group (19.3% vs. 15.6%, *P *= 0.20) [61]. Because the event rate in the low-Hb group was about half that expected, CREATE may be underpowered to detect differences in cardiovascular outcomes [62].

The differences between the CHOIR and CREATE results and those of earlier studies invite assessment of the factors underlying the differences and their implications for anemia treatment in the CKD population not requiring renal replacement therapy.

#### Cardiovascular Benefits and Risks

Data from both the CHOIR [60] and CREATE [61] studies have generated concern that Hb targets >13 g/dL are associated with increased incidence of cardiovascular complications and serious adverse events. In CHOIR, 125 endpoint events (composite of death, myocardial infarction, hospitalized CHF without dialysis, or stroke) occurred in the high-Hb group versus 97 events in the low-Hb group (HR, 1.34; 95% CI, 1.03 to 1.74; *P *= 0.03). CHOIR's surprising results echo those of an earlier prospective trial [63] in dialysis patients that was terminated early because of a trend toward higher rates of death and first non-fatal myocardial infarction with Hct targets of 42% versus 30%. Nevertheless, the lowest mortality rates in the latter study occurred in those patients with the highest Hct (32–42%).

In CHOIR [60], the lower Hb target (11.3 g/dL) was associated with a significantly higher incidence of myocardial infarction reported as an adverse event than the higher Hb target (13.5 g/dL) (10 patients [1.5%] vs 19 patients [3%], *P *= 0.05).

In CREATE [61], the high-Hb group reported a greater incidence of hypertensive episodes (89 patients [30%] vs 59 patients [20%]; *P *< 0.005) and headaches (31 patients [10%] vs 16 patients [5%]; *P *= 0.03) in comparison with the low-Hb group. These findings, together with earlier reports of hypertension in ESA-treated CKD patients [58,64-67] emphasize the need to monitor blood pressure carefully during erythropoietic treatment. Current labeling of approved agents warns against beginning anemia treatment in the presence of uncontrolled hypertension [68,69].

The currently ongoing Trial to Reduce Cardiovascular Events with Aranesp (TREAT) [70] is a double-blind study comparing darbepoetin alfa treatment (Hb target, 13 g/dL) versus placebo in patients with type 2 diabetes and CKD to assess effects on cardiovascular morbidity due to acute myocardial ischemia. Placebo recipients are eligible for a rescue darbepoetin administration only if their Hb falls below 9 g/dL. TREAT has currently enrolled 3500 of 4000 planned patients [71] – more than CREATE and CHOIR combined. Patients will be followed until the required number of endpoint events for analysis have accrued (i.e., TREAT is an event-driven study). Its Data Safety Monitoring Board recently evaluated interim results in view of CREATE, CHOIR, and the March 2007 Food and Drug Administration (FDA) advisory [72,73] and allowed TREAT to continue [71].

#### Renal Benefits and Risks

When erythropoietic agents were first introduced, animal data suggesting a potential adverse effect on renal disease progression was a focus of concern. Subsequent research linked this effect with increases in blood pressure associated with rapidly rising Hb levels, an effect that was prevented by appropriate control of Hb and blood pressure. A recent review suggests that treating anemia of CKD does not hasten progression to renal replacement [74]; indeed, some studies point to possible renoprotection (Table 2). In a retrospective study of US veterans [55], the rate of decline in kidney function (least-squares slope of the reciprocal of serum creatinine) was almost halved after the onset of epoetin use as compared with the pre-treatment rate. In a 2004 randomized study [58] comparing epoetin treatment targeted to Hb >13 g/dL with deferred treatment beginning at <9 g/dL, roughly half as many patients required renal replacement in the early group (10 of 45 patients) as in the late group (18 of 43 patients).

In CREATE [61], although the rates of eGFR change did not differ between groups, a significantly higher rate of progression to dialysis occurred in patients assigned to a high-Hb level (13 to 15 g/dL) than a low-Hb level (10.5 to 11.5 g/dL). In CHOIR [60], in contrast, proportions of patients requiring renal replacement did not differ between groups with Hb targets of 11.3 or 13.5 g/dL.

There is a paucity of information on the effect of anemia treatment on *measured *GFR. Effects of anemia treatment on renal function were assessed by disparate methods among the studies cited in Table 2 and those in the Cochrane systematic review [67]. Discordances among renal results in CREATE, CHOIR, and previous studies point to the need for a further randomized trial of anemia therapy in which change in the rate of decline in measured GFR is a primary endpoint.

#### Cognition and Quality of Life

In patients on dialysis, untreated anemia can result in objective cognitive deficits [75], and treatment of anemia is associated with improved cognitive and social functioning [76]. Thus, cognitive and quality-of-life effects have also been assessed in patients at earlier stages of CKD receiving anemia treatment. A meta-analysis in this population associates erythropoietin use with improved physical function, energy, sense of well-being, and ability to work [67]. In CREATE, mean quality-of-life scores were higher in the normalized Hb group (13.0 g/dL to 15.0 g/dL) than the low-Hb group (10.5 g/dL to 11.5 g/dL) during the first year and became similar between groups thereafter [61]. In CHOIR, quality of life did not differ between Hb target groups. Thus, improvement in quality of life with erythropoietic treatment may be intuitive but is not yet proven.

### Management of Adults with Anemia of Chronic Kidney Disease

#### Clinical Evaluation and Diagnosis

Screening for anemia and other comorbidities is essential for patients diagnosed with Stage 3 CKD [16]. The course of CKD is often gradual (years to decades), and decline in Hb, like decline in eGFR, may be evident only with periodic evaluation. Annual determination of renal function and Hb levels may suffice for slowly progressing or early CKD [16]. Patients with moderate-to-severe CKD may require more frequent Hb monitoring since the likelihood of anemia is greater in this population; more frequent monitoring (at least monthly) is also required during treatment with stimulants of erythropoiesis. Patients with an eGFR below 30 mL/min/1.73 m^2 ^are considered appropriate for referral to a nephrologist, and many PCPs and nephrologists prefer a higher eGFR referral trigger as Stage 3 approaches. It is prudent to screen for anemia in CKD patients during and after acute episodes of uncontrolled comorbid disease (eg, poor glycemic control).

All patients with independent risk factors for CKD-related anemia warrant close hematologic evaluation during follow-up clinic visits. In addition, diabetic patients are twice as likely to develop anemia as their nondiabetic counterparts at the same level of renal function [37,70], and the prevalence of anemia in patients with cardiovascular disease is also significant [42,43]. Patients should also be checked for malnutrition and vitamin deficiency syndromes.

A Hb level below 12.0 g/dL in women or 13.5 g/dL in men warrants clinical work-up for anemia (Table 3). In general, CKD-related anemia is normochromic and normocytic with bone marrow of normal cellularity. With impaired production and/or activity of erythropoietin, the anemia is usually hypoproliferative, as determined by the absolute reticulocyte count.

**Table 3:** Management of anemia in patients with chronic kidney disease (CKD) [16, 29]

**Intervention**	**Significance**
**Identification and clinical evaluation**	

Screening	Patients with CKD should be evaluated for the presence of anemia once GFR reaches 60 mL/min. Kidney function (and Hb level) should be assessed in all patients with cardiovascular disease and diabetes.

**Hematological work-up**	

Hb	Determines severity of anemia. Hb is a more reliable surrogate marker than hematocrit. Dosages of erythropoietic agents are titrated to the absolute Hb value, taking into account the relative increase from the last dosage.
Complete blood count (MCH, MCV, MCHC, white blood cell count, platelet count)	Information on: potential folate and vitamin B_12 _deficiency (high MCV indicative of macrocytosis); iron deficiency (low MCH indicative of hypochromia); and capacity of bone marrow function.
Absolute reticulocyte count	Determination of proliferative activity
Serum ferritin	Assessment of iron storage reserves (target, 200 ng/mL). There is little evidence to suggest treating patients with levels >500 ng/mL is worthwhile.
TSAT or Hb content in reticulocytes	Iron balance and distribution (TSAT target > 20%).

**Treatment**	

Target risk factors	Progression of CKD can be delayed by tight control of blood pressure, blood glucose, and proteinuria.
Stimulants of erythropoiesis	Recommended in anemic patients to maintain Hb levels between 11.0 g/dL and 12.0 g/dL. Monthly follow-up is required to ensure the regimen does not raise Hb >12 g/dL and/or induce hypertension.
Iron	Oral iron preparations (FeSO_4_, Niferex, Proferrin, etc.) may be sufficient to raise iron stores, though monthly IV iron supplementation may be required to ensure optimal erythropoiesis in patients with iron-deficiency anemia. Iron gluconate or iron sucrose are safer than iron dextran, which has been associated with anaphylaxis. Emerging IV iron agents are designed to minimize free iron and oxidative stress; an emerging oral iron agent utilizes the heme iron receptor in the gut for enhanced absorption.
Nutritional supplements	Oral supplementation of folate, pyridoxine and vitamin B_12 _(and other vitamins) is a rational choice in malnourished patients.
Androgens	Not recommended.

**MCH **= mean corpuscular hemoglobin; **MCV **= mean corpuscular volume; **MCHC **= mean corpuscular hemoglobin concentration, **Hb **= hemoglobin; **TSAT **= serum transferrin saturation.

#### Treatment

While the propriety of treating anemia of CKD is well established to within a Hb target range of 11–12 g/dL, full normalization of Hb in these patients remains controversial, and benefits remain unproven. New guidelines published by the Kidney Disease Outcomes Quality Initiative (KDOQI) in August 2007 recommend a Hb target range of 11–12 g/dL for patients with CKD [29]. The CHOIR [60] and CREATE [61] studies indicate evidence of risk and no evidence of benefit from treating to Hb levels >13.0 g/dL as compared with ≤12 g/dL. A 2007 FDA advisory [72,73] recommends maintaining Hb within the range of 10–12 g/dL. Evidence reviewed in the KDOQI guidelines [16,29] suggests that treating to maintain Hb at or above 11 g/dL provides quality of life benefits without increased adverse events. Routine monitoring (preferably monthly) of blood pressure, renal function, Hb, and iron studies is required to obtain the most effective regimen of erythropoietic therapy [68,69]. The current FDA advisory [72,73] recommends more frequent Hb monitoring (twice weekly) during initial correction of anemia and after ESA dose changes.

It is essential to control hypertension before and during stimulated erythropoiesis [72,73]. Determination of serum ferritin and transferrin saturation is advised before initiation of erythropoietic therapy and every 1–3 months during therapy (Table 3) [16]. Patients with stable Hb in the target range who are receiving a stable dose of an erythropoietic agent should have their Hb checked monthly [16]. In iron-deficient patients, oral supplementation with inorganic iron salts may be sufficient, but more often parenteral iron is required in the form of iron gluconate or iron sucrose, which have supplanted iron dextran because of superior safety profiles. Physiologic levels of folate, vitamin B_12_, and pyridoxine can be maintained with oral supplementation.

## Conclusion

Anemia, a clinical manifestation of reduced kidney function, is often underrecognized in patients with CKD. Substantial mortality and morbidity are associated with advanced CKD, and current evidence suggests that early proactive multimodal treatment can improve outcomes.

PCPs are uniquely positioned to screen at-risk patients for early CKD and anemia. In most patients, the severity of anemia can be easily reduced by use of erythropoietic agents and intravenous iron as necessary in the primary care setting. Monthly follow-up is required to evaluate general cardiorenal health and to ensure that Hb levels do not overshoot the optimal range of 11–12 g/dL, given current questions regarding the optimal Hb target. The burden of CKD and its complications is expected to continue to increase. With a shortage of nephrologists predicted, an expanded role for PCPs in the management of CKD and its attendant anemia may avert this potential public health crisis.

## Competing interests

RJS: advisor or consultant – Roche, Ortho Biotech, Amgen

CLD: speakers' bureau – Amgen

## Authors' contributions

RJS and CLD jointly participated in the article's conceptual development and multiple substantive revisions and approved the final version.
